# HOPX: A Unique Homeodomain Protein in Development and Tumor Suppression

**DOI:** 10.3390/cancers14112764

**Published:** 2022-06-02

**Authors:** Ravindran Caspa Gokulan, Lee Fah Yap, Ian C. Paterson

**Affiliations:** 1 Sylvester Comprehensive Cancer Center, Department of Surgery, University of Miami, Miami, FL 33136, USA; 2 Oral Cancer Research Coordinating Centre, Department of Oral and Craniofacial Sciences, Faculty of Dentistry, University of Malaya, Kuala Lumpur 50603, Malaysia; yapleefah@um.edu.my; 3 Oral Cancer Research Coordinating Centre, Faculty of Dentistry, University of Malaya, Kuala Lumpur 50603, Malaysia

**Keywords:** HOPX, tumor suppressor, senescence, squamous differentiation, apoptosis

## Abstract

**Simple Summary:**

Homeobox (HOX) genes encode homeodomain proteins that regulate a wide range of molecular pathways. The homeodomain is highly conserved and binds to DNA. One exception is homeodomain-only protein (HOPX) that lacks DNA-binding capacity. HOPX plays a crucial role in development and its functional impairment is associated with a variety of diseases, including cancer. Loss of HOPX function occurs in a wide range of cancer types, where it functions as a tumor suppressor gene. Understanding the molecular mechanisms by which HOPX regulates carcinogenesis will likely lead to the development of new therapeutic approaches.

**Abstract:**

Homeobox genes are master regulators of morphogenesis and differentiation by acting at the top of genetic hierarchies and their deregulation is associated with a variety of human diseases. They usually contain a highly conserved sequence that codes for the homeodomain of the protein, a specialized motif with three α helices and an N-terminal arm that aids in DNA binding. However, one homeodomain protein, HOPX, is unique among its family members in that it lacks the capacity to bind DNA and instead functions by interacting with transcriptional regulators. HOPX plays crucial roles in organogenesis and is expressed in both embryonic and adult stem cells. Loss of HOPX expression is common in cancer, where it functions primarily as a tumor suppressor gene. In this review, we describe the function of HOPX in development and discuss its role in carcinogenesis.

## 1. Introduction

Homeobox genes are master regulators of morphogenesis and cell differentiation. All such genes contain a “homeobox”, which is a DNA sequence of roughly 180 base pairs that encodes the homeodomain, which comprises three helical regions folded into a tight globular structure that binds a 5’-TAAT-3’ core motif. The homeodomain is highly conserved and is identical from flies to humans [1,2] and the 60-amino-acid motif is responsible for binding to target genes to activate transcription [1,3,4]. As transcription factors, homeodomain proteins are involved in embryonic patterning and cell differentiation, and many have been connected to human diseases [5]. Based on the percentage of sequence similarity, homeobox genes are classified into several families, such as HOX, NKX, PAX, GBX2, and MSX [6]. Different functional properties amongst families are determined by DNA-binding specificities, co-factors, and other mechanisms, such as additional domains or motifs found within homeoprotein families [6].

Homeobox genes are necessary for diverse biological functions as they control the expression of a wide range of genes that play crucial roles in proliferation, differentiation, survival, cell cycle, migration, and apoptosis. Consequently, there are many target genes with a variety of known functions, and these include p53, p21, VEGF, osteopontin, IGFBP1, NCAM, keratins, and integrins [1]. The different homeodomain families possess varied biological functions. For example, MSX genes regulate epithelial to mesenchymal transition during organogenesis [7], whereas members of NKX, PAX, and DLX are involved in differentiation in a tissue-specific manner [6]. Homeodomain proteins are not specific to adult differentiated cells but are also seen in adult stem cells, such as CD34+ hematopoietic stem cells [8]. In particular, the crosstalk between embryonic- and differentiation-specific homeodomain proteins provides a link between organogenesis and cancer.

Not surprisingly, the expression of homeobox genes is often altered in human diseases, with certain homeobox genes being upregulated while others are downregulated. For example, the homeobox gene Oct-4, which is associated with embryonic stem cells [9], has been found to be upregulated in prostate cancer whilst the homeobox gene NKX3.1, on the other hand, has been shown to be downregulated [10]. Collectively, homeobox genes play critical roles in inducing oncogenic transformation in vitro by modifying a range of biological processes [8].

Whilst the majority of homeodomain proteins bind DNA and function as transcription factors, one related protein, HOPX, is unusual in that it lacks DNA-binding capability and functions only as a transcriptional regulator. Here, we will review our current understanding regarding the function of HOPX in development and its role in carcinogenesis. 

## 2. HOPX Structure

HOPX was initially identified by two independent research groups through the screening of EST databases for novel homeobox genes expressed in the developing mouse heart [11,12]. HOPX has also been termed Toto, Cameo, Ob1, SMAP31, NECC1, and LAGY in the published literature [13,14,15]. Subsequently, the HOPX gene symbol was given to homeodomain-only protein (HOP) to differentiate it from other genes such as hopscotch in Drosophila and hop-sterile in mouse. HOPX belongs to the PRD class of homeodomain proteins [16,17].

**Figure 1:**
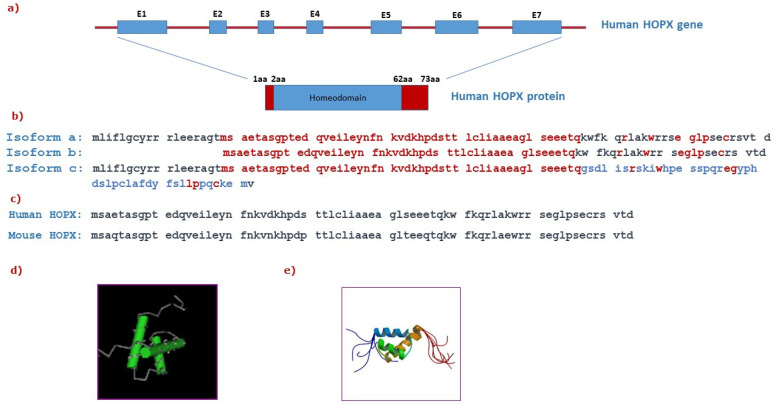
Structure of the HOPX gene and protein: (**a**) Graphical representation of the human HOPX gene containing seven exons (E1-E7) and intronic sequences (depicted as a red thick line) encoding for a 73aa protein. (**b**) Comparison of the amino acid sequences between human HOPX protein isoforms a, b, and c. The common sequence in all three isoforms is highlighted in red. The amino acids that are only found in isoform c are shown in blue, indicating a distinct C-terminal sequence. (**c**) Comparison of the amino acid sequences between human and mouse HOPX proteins. (**d**) Graphical representation of the homeodomain that is conserved among homeodomain proteins and its respective sequence (adapted from NCBI). (**e**) The solution structure of HOPX (adapted from RCSB PDB).

The human HOPX gene, located on chromosome 4q12, encodes a nuclear protein [11,12] and primary sequence analysis and secondary structural prediction indicates that HOPX is closely related to members of the homeodomain family and forms three ∝ helices, with a helix-turn-helix motif characteristic of the homeodomain [12] (Figure 1). HOPX contains the 60-amino-acid homeodomain but has a number of unusual characteristics that distinguish it from other known homeodomain proteins [11,12] (Figure 1). HOPX forms a classical homeodomain fold and a single amino acid insertion between helix I and II, but it lacks DNA-binding capacity due to the absence of several key residues known to mediate critical contacts with DNA that are conserved among other homeodomain proteins [11,12,18] (Figure 1). Exons 1, 5, 6, and 7 of the HOPX genes are alternatively spliced to generate five mRNA transcripts. These transcripts encode three protein isoforms: isoform a (previously γ) consists of 91 amino acids, isoform b (previously β) contains 73 amino acids, and isoform c (previously α) is longer with 112 amino acids, giving a different C-terminal sequence [19]. Only the HOPX b (β) promoter harbors CpG islands, which is relevant to the HOPX silencing observed in cancers discussed later. The precise function of the individual splice variants and protein isoforms remains to be determined. 

## 3. HOPX in Development and Differentiation

Homeodomain-containing proteins act as transcription factors/regulators to regulate cell differentiation and developmental processes, and their target genes are diverse and complex [6]. In this regard, HOPX expression has been detected in both embryonic and adult stem cells in various tissues to regulate crucial biological processes.

### 3.1. Embryonic Stem Cells

HOPX is expressed during embryonic development of skeletal muscle, stratified epithelium (upper digestive tract and skin), epithelium of developing airways, midbrain/hindbrain junction, meninges, cartilage, chondrocytes, and lens fiber cells [13,20]. HOPX also has a role in placental development, controlling transcription factors in concert with other homeobox genes. HOPX expression has been detected in the trophoblast stem cells of E8.5–E9.5 wild-type mouse placentas [21]. The syncytiotrophoblast of humans, an epithelial layer of the highly vascular embryonic placental villi, which invades the uterine wall to create nutritional circulation between the embryo and the mother, expresses HOPX. In response to differentiation stimuli, HOPX induces the differentiation of trophoblast stem cells into a trophoblastic lineage [21]. HOPX regulates the development of endothelial cells, thereby contributing to primitive hematopoiesis by at least partially inhibiting Wnt signaling [22].

### 3.2. Adult Stem Cells

HOPX expression has been detected in various adult stem cells such as bone marrow, intestine, hair follicles, and lung [23,24,25]. HOPX is crucial for the proper maintenance of intestinal epithelium. There are two stem cell niches within the intestinal epithelium: one at the +4 position that contains slow-cycling and label-retaining cells and the other at the crypt base with crypt base columnar (CBC) cells. The cells at the +4 position have a unique transcriptional profile, which includes the expression of HOPX [26,27]. These +4 cells have previously been described as part of the resting stem cell population, which rebuilds Lgr5+ CBCs upon injury and has been found to be radiation resistant, implying that they can survive crypt injury and replace their damaged counterparts [28]. Furthermore, HOPX-positive intestinal stem cells in the +4 locations are resistant to anoikis, a type of apoptosis caused by aberrant cell matrix connections [29]. HOPX-positive stem cell populations have also been shown to express CD24 and CD44 [30]. 

Recently, the epigenetic factor, enhancer of zeste homolog 2 (EZH2), was found to interact directly with the HOPX promotor regions during bone marrow stromal cell (BMSC) differentiation to promote osteogenesis [31]. The telogen basal bulge’s resident stem cells of hair follicles have also been found to express HOPX. These HOPX^+^ hair follicle cells perform a function in epidermal wound repair in addition to differentiating into all lineages. In the lower hair bulb of the anagen phase, an HOPX+/Lgr5+/Shh- progenitor population was identified; when these cells differentiate into k6-positive inner bulge cells in telogen, they govern nearby hair follicle stem cells [24].

The type I cells of the mouse lung epithelium, which maintain gas exchange in alveoli, are HOPX-positive. Despite undergoing proliferation, these HOPX-positive type I cells give rise to type II cells upon partial pneumonectomy-stimulated alveolar regeneration in mice [32]. Furthermore, during the differentiation of mouse splenic marginal zone precursors (MZPs) into marginal zone B (MZB) cells caused by the Notch 2 signaling cascade, HOPX was found to be downregulated [33]. HOPX is a stem cell marker that is also associated with stem cell features. Zhou et al. identified a significant association between HOPX and hematopoietic stem/progenitor cell (HSPC) frequency by using genome-wide association studies (GWASs) to uncover genetic determinants [34]. In mice, hematopoietic cell-specific knockout of HOPX causes stemness and quiescence to be disrupted [35]. 

In adult pancreatic tissues, pan-islet cells are positive for HOPX, and they may play a possible role in maintaining endocrine cell identity [36]. Several lines of evidence have suggested that HOPX functions as a negative cell cycle regulator. High levels of HOPX have been found in the inner trabecular layer of the heart during mouse development, where cell proliferation is lower than in the outer compact zone, and HOPX appears to be essential for appropriate cell cycle withdrawal in the perinatal period [11,12]. A number of genes involved in cell proliferation were found to be elevated in the heart tissues of HOPX-null mice [12]. HOPX has also been demonstrated to control the balance between proliferation and differentiation in the growing heart and cells of the central nervous system [12,37]. HOPX regulates apoptosis of radial astrocytes in the dentate gyrus of the mouse hippocampus, which are required for the formation of hippocampal granular neurons throughout life [38]. 

## 4. HOPX Regulation of Molecular Signaling Networks

HOPX functions as a mediator of multiple signaling pathways in both normal and pathological conditions. HOPX has been identified as a direct downstream transcriptional target of Nkx2.5, a cardiac-specific transcription factor, in a series of in vitro and in vivo experiments in mice [11,12,39]. During cardiac development, HOPX directly binds with the serum response factor (SRF) to enhance its detachment from DNA, thereby inhibiting SRF-mediated transcription. HOPX also interferes with SRF’s cooperation with its coactivator [11,12] and can inhibit SRF-dependent transcriptional activation by recruiting histone deacetylases (HDACs) such as HDAC2 [40]. Two distinct regions on the surface of the HOPX protein are thought to be needed for its capacity to interact with other proteins, such as SRF and HDAC, to control transcription rather than binding to DNA [18]. Moreover, because it lacks intrinsic DNA-binding capability, HOPX interacts with transcription activators or repressors [11,12,40]. The association between HOPX and HDAC2 was further strengthened by the co-expression of these two proteins in developing pulmonary airway epithelium, where HOPX functions as a HDAC-dependent negative regulator of surfactant protein development. During pulmonary maturation, HOPX and HDAC act as downstream regulators of Nkx2-1 and GATA6 [41]. 

HOPX interacts with SRF and prevents the survival of dentate gyrus progenitor cells by inhibiting the activation of SRF target genes such as Bcl2 [38]. However, HOPX expression has been demonstrated to be spatially and temporally controlled in the developing central nervous system, even at sites where SRF was not present, suggesting that HOPX’s role in neurogenesis is distinct from that of cardiogenesis [37]. HOPX promotes cardiomyogenesis by fostering the differentiation of cardiomyoblasts into cardiomyocytes, interacting with activated Smads of the BMP signaling cascade and inhibiting Wnt signaling [42]. In addition to SRF and HDAC, HOPX interacts with enhancer of polycomb 1 (Epc 1), which is highly expressed in the embryonic heart and skeletal muscles. A physical interaction between HOPX and Epc 1 was found in yeast and mammalian cells. For example, myogenin is activated and myotube formation is stimulated in H9c2 cells following co-transfection of HOPX and Epc 1, and Epc 1-mediated skeletal muscle differentiation was disrupted in HOPX-knockout mice [43]. 

Lee et al. showed a significant downregulation of HOPX during the transdifferentiation of myogenic satellite cells (MSCs) into adipocyte-like cells (ALCs) [44]. HOPX has also been demonstrated to be required for keratinocyte differentiation, and it is controlled by a number of signaling pathways following cell cycle exit, including PKC, phospholipase C, AKT, and NFĸB. Interestingly, the activation of HOPX during keratinocyte differentiation is dependent on the PKC pathway rather than DNA methylation [45].

The role of HOPX in T cell lineages is distinct from that of other cell types. Higher levels of HOPX expression were seen only in terminally differentiated T-helper type I (Th 1) cells and not in naïve T-helper (Th) cells. The AP-1 complex, which includes c-Fos and c-Jun, is well known for maintaining cell proliferation. By inactivating c-Fos, which is controlled by SRF, HOPX modulates T cell proliferation and antigenic responses and enables T cell antigen unresponsiveness [46]. HOPX, in particular, suppresses Fas-induced apoptosis, and only HOPX-positive murine Th 1 cells persist in vivo [47]. Jones et al. found a negative association between HOPX and IL-2 in peripheral regulatory T cells (pTregs), which protects pTregs against autoimmune responses to self-antigens [48]. 

It has been demonstrated that altering normal HOPX function causes significant developmental heart abnormalities in mouse and zebrafish models [11,12,40]. HOPX expression is reduced by the SRF- and GATA4-dependent pro-hypertrophic response, and it is completely absent in severe heart failure [49]. During HOPX-induced hypertrophy in rats, inhibition of histone deacetylase (HDACi) is critical for atrial fibrosis and arrhythmic inducibility [50]. Two variants in the HOPX gene have been shown to cause syncope, which is defined as a loss of consciousness and muscle strength in patients with hypertrophic cardiomyopathy (HCM) [51].

**Figure 2:**
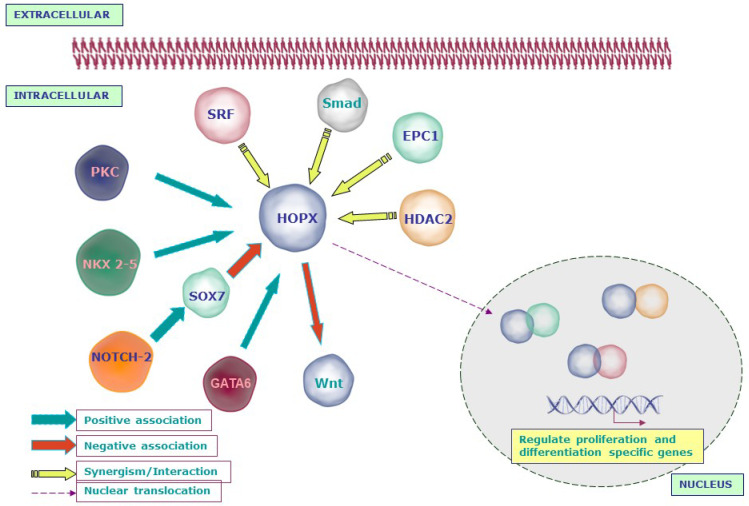
Schematic representation of the molecular networks regulated by HOPX in development: HOPX is activated by the signaling molecules that are essential for normal development such as NKX2-5, PKC, and GATA6. However, NOTCH2 inhibits HOPX through SOX7. HOPX interacts with serum response factor (SRF) and inhibits SRF-mediated transcription. On the other hand, HOPX recruits histone deacetylase 2 (HDAC2) to modulate the function of SRF. Apart from SRF and HDAC, HOPX interacts with enhancer of polycomb 1 (Epc-1) to activate myogenin and induces myotube formation. During cardiomyogenesis, HOPX and Bmp signals synergistically inhibit Wnt signaling through activated Smads.

The molecular mechanisms of HOPX during development are summarized in Figure 2.

## 5. HOPX in Carcinogenesis

### 5.1. HOPX as a Tumor Suppressor Gene

There is now a wide body of evidence, which we outline below, that indicates that HOPX functions to suppress the carcinogenic process. The expression, methylation status, and function of HOPX in different cancer types is summarized in Table 1.

#### 5.1.1. HOPX Expression in Normal and Malignant Tissues

HOPX was initially isolated using a PCR-based subtracted fragmentary cDNA library between normal placental villi and a choriocarcinoma cell line [14]. HOPX expression was later detected in normal tissues of the lung, smooth muscle, brain, placenta, bladder, spleen, and kidney [14]. Subsequently, HOPX was shown to be downregulated in a wide range of human tumor types, including lung cancer [15], choriocarcinoma [14], head and neck cancers [52,54,63,64,65,66], esophageal squamous cell carcinoma [53], glioblastoma [38], human uterine endometrial cancers (HECs) [59], gastric cancer [55], colorectal cancer [60,61], and pancreatic cancer [62], suggesting a tumor suppressor role for HOPX. 

More direct evidence for a tumor suppressor role for HOPX has come from in vivo studies, where ectopic expression of HOPX in choriocarcinoma [14], esophageal [53], glioblastoma [38], HEC [59], gastric [55], colorectal [61], and lung cancer [56] cells resulted in decreased tumor growth suppression in vivo. Interestingly, loss of HOPX expression has clinical relevance, at least in lung cancer, because decreased HOPX expression in lung SCC has been linked to high-grade and advanced-stage cancers [15] and miR-421 has been shown to target HOPX, which then stimulates the Wnt/catenin signaling pathway, promoting the development of non-small cell lung cancer [67].

Despite its function as a tumor suppressor, HOPX gene mutations appear to be rare in cancer [59]. The precise role of the individual splice variants is still unknown; however, all variants were detected in HNSCCs [66], and in skin cancer [68] and acute myeloid leukemia [69], the level of transcripts 1, 3, and 5 were reported to be low while variants 2 and 4 were predominantly expressed. 

#### 5.1.2. HOPX Promoter Methylation

Methylation of the cytosine residues of the CpG island within the promoter region of tumor suppressor genes is one of the mechanisms that suppresses their function in cancer [70]. HOPX promoter methylation has been detected in human uterine endometrial cancer [59], head and neck cancer [66], gastric cancer [55], colorectal cancer [60,61], pancreatic cancer [62], and lung cancer [56]. Methylation of the HOPX promoter can occur as an early event in carcinogenesis because it has been detected in the early stages of gastric cancer [55]. Interestingly, HOPX has been ranked second among the top priority genes that undergo methylation in primary esophageal squamous cell carcinoma [53]. Treatment of head and neck, esophageal, and pancreatic cancer cells with demethylating drugs such as 5-aza-2-deoxycytidine restored HOPX expression [53,62,66]. HOPX does not harbor mutations in human uterine endometrial cancer; however, its hypermethylation demonstrates epigenetic modification as a crucial event for inactivation [59]. Loss of HOPX expression as a result of promoter hypermethylation has clinical significance because in patients with gastric and colorectal cancer, the methylation status has been linked to a worse prognosis [55,61] and patients with strong HOPX expression in lung cancer have a considerably longer survival [56]. Epigenetic suppression of HOPX by promoter methylation plays a critical role in aggressive phenotypes in papillary thyroid cancer and is correlated with a poor prognosis of patients [71].

#### 5.1.3. HOPX Affects Tumor Cell Behavior

**Figure 3:**
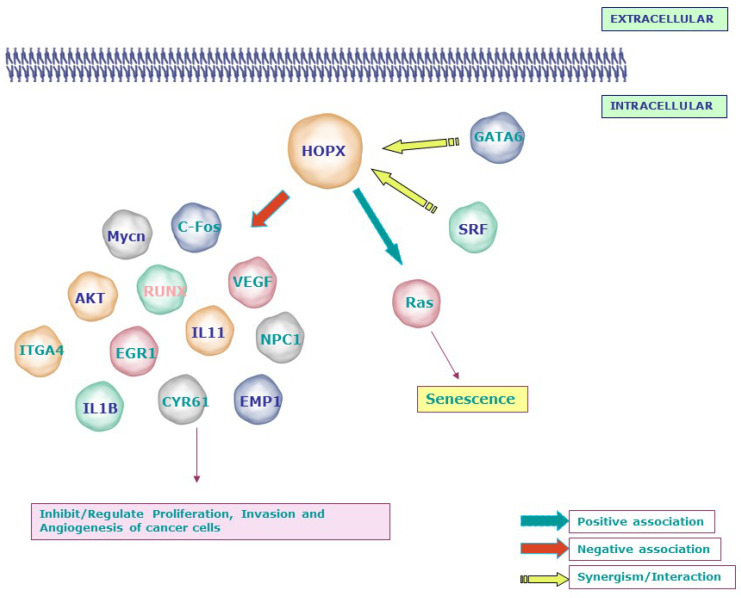
Signaling molecules related to the tumor suppressor function of HOPX: Loss or gain experiments and microarray analyses revealed the negative association between HOPX and molecules involved in invasion, angiogenesis, and transformation, including Mycn, c-Fos, AKT, VEGF, EGR1, IL11, IL1B, and EMP1. In line with its mechanism during development, HOPX affects the proliferation of cancer cells by synergizing with SRF and GATA6. Notably, HOPX induces senescence through the RAS-ERK-MAPK signaling cascade in lung cancer. SLC2A3—Solute Carrier Family 2 (Facilitated Glucose Transporter), Member 3; NPC1—Niemann–Pick disease, type C1; RUNX1—Runt-related transcription factor 1; CNN1/CYR61—Cysteine-rich, angiogenic inducer, 61; WTAP—Wilms tumor 1-associated protein; PRDX2—Peroxiredoxin 2; EREG—Epiregulin; ITGA4—Integrin, alpha 4; EMP1—Epithelial membrane protein 1; EPHA2—EPH receptor A2; EGR1—Early growth response; MYCN—v-myc avian myelocytomatosis viral oncogene neuroblastoma-derived homolog; IGFII—Insulin-like growth factor 2 (somatomedin A).

Numerous in vitro and in vivo studies have shown that HOPX affects a variety of cellular processes and loss of HOPX promotes the malignant phenotype of tumor cells by a variety of mechanisms, as summarized below. The possible signaling networks regulated by HOPX in cancer are depicted in Figure 3.

HOPX was found to be involved in the regulation of the cell cycle and apoptosis. By positively regulating the subG1 and GO/G1 phases, HOPX reduces DNA synthesis and promotes cell death in pancreatic cancer [62]. Glioblastomas (GBMs) are derived from neural cancer stem cells and HOPX regulates the hippocampal stem cell niche through controlling cell survival and apoptosis, but it is downregulated in GBMs in mice; a key finding of this work is that reactivating HOPX causes apoptosis and lowers the tumorigenic ability of GBM cancer stem cells [38]. HOPX is epigenetically silenced in breast cancer. HOPX overexpression causes apoptosis and cell cycle arrest in breast cancer cells, suggesting that it could be utilized as a therapeutic target for breast cancer patients [72]. 

HOPX is activated by 17b-estradiol (E2) and inhibits cell proliferation in vitro and in vivo, suppressing uterine endometrial malignancies (HEC) [59]. This occurs by influencing the activity of SRF and the expression of its targets, c-Fos and Cyclin D1. HOPX activity was restored when HEC cell lines were treated with the histone deacetylase inhibitor TSA and inhibiting HOPX also stimulated immortalized human endometrial cells to proliferate [59]. In esophageal squamous cell carcinoma, however, HOPX had no effect on SRF activity, showing that the tumor suppressor functions of HOPX are cell type dependent [53]. DNA microarray analysis in colorectal cancer cells showed that the re-expression of HOPX upregulates WTAP and PRDX2 while downregulating genes associated with proliferation, angiogenesis, and invasion both in vitro and in vivo [61]. 

HOPX expression has been reported to be reduced at both the DNA and protein levels of oral and nasopharyngeal cancer cells, and its re-expression promoted cell proliferation and invasion and made the cells more susceptible to UV and cisplatin-induced cell death [66]. 

Interestingly, a recent study showed that the HOPX knockdown in squamous cell carcinoma (SCC) cells reduces their proliferative and invasive capabilities through the acceleration of apoptosis. The methylation status of two distinct HOPX promoters, promoter 1 and 2, causes the HOPX transcript expression to differ in normal keratinocytes and SCC cells. The activation of the second promoter of HOPX may contribute to the pathogenesis of SCC [68]. 

#### 5.1.4. HOPX and Senescence

There are a limited number of reports suggesting a role for HOPX in cellular senescence. For example, HOPX transfection of lung cancer cells resulted in enlarged and flattened morphologies, with positive SA—Gal staining indicating senescence, and stimulated the expression of proteins associated with aging, such as SAHFs and p-H2AX. When these cells were treated with senescence-inducing drugs such as Adriamycin (ADR) and H_2_O_2_, senescence was caused in HOPX-transfected cells but not in mock transfectants [56]. In H2228 lung adenocarcinoma cells, however, HOPX did not cause senescence, showing that it is involved in oxidative stress and DNA damage-induced cellular senescence but does not cause senescence on its own [56].

It was reported that re-expressing HOPX in immortalized bronchial epithelial cells promotes senescence. The RAS-ERK-MAPK signaling cascade has been identified to be involved in HOPX-induced senescence in lung cancer cells. A significant reduction of AKT/MDM2 accompanied by an upregulation of P53/ P21 was observed in HOPX-transfected lung cancer and normal bronchial epithelial cells. This study revealed HOPX as a regulator of the expression of senescence mediators [56]. Furthermore, the HOPX gene was shown to be overexpressed in keratinocytes with shortened telomeres, which elicits senescence [73].

#### 5.1.5. HOPX in Cancer Cell Metastasis

A critical molecular event in the development of lung cancer is the downregulation of HOPX and overexpression of Mycn [74], and re-expression of HOPX suppresses lung cancer cell proliferation migration and invasion [56]. Furthermore, the alveolar lineage transcription factors, GATA6 and HOPX, were reported to be essential in airway epithelial specification and lung adenocarcinoma subtype metastasis inhibition [57].

In a chicken metastatic cell line derived from v-src-induced sarcoma and its non-metastatic subclone, the role of HOPX in metastasis was investigated. In v-src-transformed cells, knocking down HOPX reduced metastatic activity by inactivating metastatic-associated genes such as ITGA4 [75]. The expression of EMT-related molecules, such as ZEB1/2, TWIST1, E-cadherin, and vimentin, however, was unaffected by GATA6 and HOPX knockdown. It also had no effect on Wnt or focal adhesion kinase (FAK), although the expression of src and integrin 5, two mediators of metastasis, was upregulated.

HOPX hypermethylation promotes metastasis in nasopharyngeal cancer by increasing SNAIL transcription, which facilitates epithelial to mesenchymal transition [76]. Additional metastasis-related molecules (IL1B, IL11, and EREG) and angiogenesis and ECM remodeling genes (VEGFA and PLAU) were upregulated when HOPX was knocked down [57].

**Figure 4:**
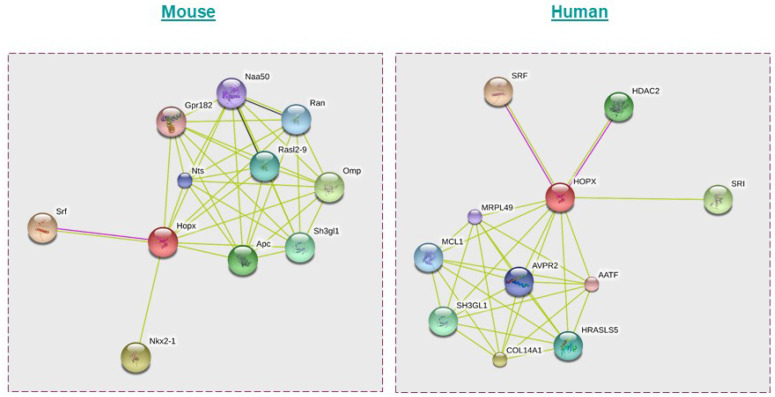
Possible interaction partners of HOPX in mice and humans as predicted using the STRING database. Apart from SRF, HDAC2, and Epc1, HOPX could interact with proteins such as Apc, Nts, Ran, and Rasl2-9 in mice and COL14A1, SH3GL1, MRPL49, and HRASLS5 in humans. **Mouse:** Nkx2-1—NK2 homeobox 1; Ran—RAN, member RAS oncogene family; Naa50—N(alpha)-acetyltransferase 50, NatE catalytic subunit Gene; Nts—neurotensin; Srf—serum response factor; Sh3gl1—SH3-domain GRB2-like 1; Gpr182—G protein-coupled receptor 182; Rasl2-9—RAS-like, family 2, locus 9; Hopx—Homeodomain-only protein; Omp—olfactory marker protein; Apc—adenomatosis polyposis coli. **Human:** SRI—sorcin; HDAC2—histone deacetylase 2; COL14A1—collagen, type XIV, alpha 1; HOPX—Homeodomain-only protein; SH3GL1—SH3-domain GRB2-like 1; SRF—serum response factor (c-fos serum response element-binding transcription factor); AVPR2—arginine vasopressin receptor 2; MRPL49—mitochondrial ribosomal protein L49; MCL1—myeloid cell leukemia sequence 1 (BCL2-related); HRASLS5—HRAS-like suppressor family, member 5; AATF—apoptosis antagonizing transcription factor.

Bioinformatic analysis using STRING software predicts the possible interacting partners of HOPX in mice and humans as indicated in Figure 4 [77].

### 5.2. Evidence for an Oncogenic Function for HOPX

The expression level of HOPX is decreased in most cancer types and it functions as a tumor suppressor. However, HOPX expression has been reported to be elevated in a small number of esophageal carcinoma cell lines (TE2, TE5, KYSE410, and KYSE520), lung adenocarcinoma, and pancreatic cancer Langerhans islet cells [53,56,62]. Strong HOPX expression was associated with specific clinical and biochemical characteristics in de novo acute myeloid leukemia and predicted a poor prognosis [69]. HOPX is one of a group of genes, including deoxynucleotidyl transferase (DNTT) and B cell liners (BLNK), that are upregulated in patients with RUNX-1 mutant cytogenetically normal acute leukemia [78]. Interestingly, HOPX expression is increased in HPV 16- and 18-infected stage IB-IIA cervical cancer cells [79]. The expression of HOPX was also noticed in the tumor microenvironment; in particular, HOPX expression was higher in normal stromal cells than in stromal cells from colorectal malignant tissue [60,61]. There is increasing evidence that HOPX is a tumor suppressor gene in numerous cancer types. However, the function of HOPX in cancer could well be context dependent and more studies are needed to confirm a possible oncogenic role for HOPX in certain circumstances.

## 6. Conclusions and Future Perspectives

HOPX is a unique homeodomain protein that does not bind DNA but rather interacts with other proteins to function as a transcriptional regulator in development. During organogenesis, HOPX regulates the differentiation of various cell types, including keratinocytes, skeletal muscle, cardiomyocytes, and chondrocytes. In cardiac tissues, HOPX performs its function by directly controlling SRF and additional roles unrelated to SRF have been described. However, the entirety of the signaling networks associated with HOPX and organogenesis has yet to be uncovered and further studies are needed to fully elucidate the critical role of HOPX in organogenesis and tissue homeostasis.

HOPX is crucial to the prevention of tumor development and progression by promoting tumor suppression mechanisms. In malignancies of the head and neck, stomach, colon, pancreas, lung, placenta, breast, and esophagus, the HOPX promoter is hypermethylated and its role as a tumor suppressor gene was proven in a variety of functional studies. In the future, it is important to understand whether these findings can be utilized in a therapeutic context. Approaches to activate downstream signaling pathways that are compromised following HOPX loss could reverse some of the effects of HOPX loss in tumor cells. Similarly, the re-expression of epigenetically silenced HOPX with demethylating drugs would likely restore its tumor suppressive functions, as demonstrated in vivo in mouse models. A fuller understanding of the molecular mechanisms by which HOPX regulates carcinogenesis will likely lead to the development of new therapeutic approaches.

## Figures and Tables

**Table 1:** Deregulation and functional significance of HOPX in cancer.

Type of Cancer	Expression Pattern and Methylation Status	Functional Insights	References
Head and Neck cancer	Hypermethylated and downregulated.	Regulates apoptosis and tumorigenesis (in vitro).	[52,53,54]
Esophageal cancer	Hypermethylated and downregulated.	Regulates tumorigenesis (in vitro) and has prognostic value. It does not affect SRF activity.	[53]
Gastric cancer	Hypermethylated and downregulated. Methylation was identified in the early stage of cancer.	Regulates proliferation, apoptosis, invasion, and tumorigenesis (in vitro). It has prognostic significance.	[55]
**Lung Cancer**	Hypermethylated and downregulated.	Regulates proliferation, apoptosis, invasion, metastasis, and tumorigenesis (both in vitro and in vivo). It induces senescence through the RAS/MAPK pathway. HOPX synergizes with GATA6 to regulate metastasis and angiogenesis of lung cancer cells. Downregulation of HOPX correlates with the differentiation status and relapses.	[15,56,57,58]
Uterine cancer	Hypermethylated and downregulated.	It partially affects the SRF-c-Fos-Cyclin D1 pathway.	[14,59]
Colon cancer	Hypermethylated and downregulated. Normal stromal cells showed high levels of HOPX expression, and it was low in cancer cells.	Regulates proliferation, apoptosis, angiogenesis, invasion, and tumorigenesis (both in vitro and in vivo). It has prognostic significance.	[60,61]
Pancreaticcancer	Hypermethylated and downregulated. Strong expression was observed in Langerhans islet cells and very weak/negative expression in pancreatic cancer cells.	Regulates proliferation, apoptosis, invasion, and tumorigenesis (in vitro). No prognostic value.	[62]
Breast cancer	Reduced expression.	Regulates proliferation and tumorigenesis (in vivo).	[59]
Placenta (Tropho)	Reduced expression.	Regulates tumorigenesis (in vivo).	[14]
Glioblastoma	Expression was reduced. Radial astrocytic stem cells showed positive staining for HOPX.	Regulates the apoptosis and tumorigenicity of glioblastoma cancer stem cells.	[38]
